# Higher Plasma Pentraxin-3 Level Predicts Adverse Clinical Outcomes in Patients With Coronary Artery Disease: A Meta-Analysis of Cohort Studies

**DOI:** 10.3389/fcvm.2021.726289

**Published:** 2022-01-10

**Authors:** Kejun Ding, Zhewei Shi, Caizhen Qian, Xuan Yang

**Affiliations:** ^1^Department of Cardiology, Zhuji Affiliated Hospital of Shaoxing University, Zhuji, China; ^2^Departments of Cardiology, Qingdao Municipal Hospital, Qingdao, China

**Keywords:** coronary artery disease, mortality, major adverse cardiovascular events, pentraxin-3, meta-analysis

## Abstract

**Background:** Association between plasma pentraxin-3 (PTX-3) and clinical outcomes in patients with coronary artery disease (CAD) remains not fully determined. An updated meta-analysis of cohort studies was performed to systematically evaluate the association.

**Methods:** Cohort studies evaluating the association between plasma PTX-3 and adverse outcomes [mortality and major adverse cardiovascular events (MACEs)] in adults with CAD were identified by systematic search of PubMed, Embase, and Web of Science databases. Only studies with multivariate analysis were included. A random-effects model incorporating the potential intrastudy heterogeneity was used for the meta-analysis.

**Results:** A total of 16 studies including 11,007 patients were included. Pooled results showed that patients with highest level of PTX-3 were independently associated with higher risk of mortality [adjusted risk ratio (RR): 2.09, 95% CI: 1.60 to 2.74, *p* < 0.001; *I*^2^ = 50%] and MACEs (adjusted RR: 1.80, 95% CI: 1.43 to 2.28, *p* < 0.001; *I*^2^ = 49%). Subgroup analyses showed that the associations between PTX-3 and poor prognosis in CAD were consistent in patients with ST-segment elevation myocardial infraction, non-ST-segment elevation acute coronary syndrome, and stable CAD (*p* < 0.05 for each subgroup). Besides, the association between PTX-3 and increased incidence of mortality and MACEs were consistent in short-term (within 1 year) and long-term (over 1 year) studies and in studies with or without adjustment of C-reactive protein (CRP) (*p* < 0.05 for each subgroup).

**Conclusion:** Higher plasma PTX-3 is associated with poor prognosis in patients with CAD, which may be independent of the CAD subtype, follow-up durations, and adjustment of CRP.

## Background

Currently, coronary artery disease (CAD) remains one of the leading causes of morbidity and mortality of the global population (1). Clinically, CAD could be categorized as stable CAD and acute coronary syndrome (ACS) and the latter could be further classified as ST-segment elevation myocardial infarction (STEMI) and non-ST-segment elevation ACS (NSTE-ACS) (2, 3). With the intensive medical care for patients with CAD, the clinical outcomes of these patients have been improved, particularly with the timely application of revascularization (4, 5). However, identification of novel risk factors for poor prognosis in patients with CAD is still of clinical significance (6). Pentraxin-3 (PTX-3) is a multimeric acute-phase inflammatory protein, which could be synthesized by various cells (7). It has been suggested that the plasma level of PTX-3 may reflect the degree of systemic inflammation (8) and endothelial dysfunction (7). Since the key pathological feature of CAD is vascular inflammation-related coronary atherosclerosis, higher PTX-3 has been related with the severity of CAD, which highlights the hypothesis that higher PTX-3 may become a predictor of poor prognosis of patients with CAD (9, 10). For example, in patients with STEMI, PTX-3 has been associated with increased coronary plaque vulnerability (11) and severe ventricular remodeling after revascularization (12). Moreover, in patients with stable CAD, peak PTX-3 values were also found to be correlated with plaque characteristics of the coronary arteries (13). However, studies evaluating the association between PTX-3 and prognosis in patients with CAD showed inconsistent results (13–28). An early meta-analysis including cohort studies before 2018 showed that elevated circulating PTX-3 may be associated with poor prognosis in patients with ACS, but not in patients with stable CAD (29). However, only 2–5 datasets were included for each outcome, and the authors mentioned that interpretation of these findings should be done with caution due to the small number of studies analyzed (29). Besides, the authors were also unable to determine whether other study characteristics could affect the potential predictive efficacy of PTX-3 such as follow-up durations and adjustment of C-reactive protein (CRP), a well-known inflammatory indicator of CAD (29). Since then, many cohort studies of a similar field have been published (13, 24–28). Accordingly, we aimed to perform an updated meta-analysis to systematically evaluate the potential prognostic role of PTX-3 in CAD, multiple predefined subgroup analyses were also performed to evaluate whether the outcome differed according to the subtype of CAD, follow-up duration, and with or without adjustment of CRP.

## Methods

The meta-analysis was performed in accordance with the Meta-analysis of Observational Studies in Epidemiology (MOOSE) (30) and the Cochrane Handbook (31) guidelines.

### Literature Search

Studies were identified *via* systematic search of electronic databases of PubMed, Embase, and Web of Science *via* the combination of the following terms: (1) “pentraxin-3” or “pentraxin 3” or “PTX-3” and (2) “coronary artery disease” or “angina” or “myocardial infarction” or “acute coronary syndrome” or “percutaneous coronary intervention” or “major adverse cardiovascular events” or “CAD” or “STEMI” or “NSTEMI” or “ACS” or “AMI” or “PCI.” This extensive search strategy was used to avoid missing of possible studies. The search was limited to human studies published in English. The reference lists of related original and review articles were also analyzed using a manual approach. The final literature search was performed on April 5, 2021.

### Study Selection

The inclusion criteria for the studies were: (1) cohort studies published as full-length articles; (2) included adults with confirmed diagnosis of CAD; (3) evaluated the association between plasma PTX-3 and risks of adverse outcomes [mortality and/or major adverse cardiovascular events (MACEs)] during follow-up duration; and (4) reported risk ratios (RRs) comparing the incidence of the above outcomes between patients with the highest and lowest level PTX-3, after adjusting multiple confounding factors, at least for age and sex. Definition of MACEs was in accordance with the criteria used in the original articles, which was defined as a composite outcome including cardiac death, nonfatal myocardial infarction, heart failure, or coronary revascularization. If studies with overlapped patients were retrieved, those with a larger sample size were included. If studies reporting outcome data with variable follow-up durations were identified, outcomes with longer follow-up durations were included. Reviews, editorials, preclinical studies, cross-sectional studies, and studies irrelevant to the aim of the current meta-analysis were excluded.

### Data Extracting and Quality Evaluation

Literature search, data extraction, and quality assessment of the included studies were performed according to the predefined inclusion criteria by two independent authors. Discrepancies were resolved by consensus. The extracted data included: (1) name of first author, publication year, and country where the study was performed; (2) study design characteristics (prospective or retrospective and sample size of each study); (3) characteristics of the patient including the diagnosis of the patients, age, sex, and proportions of patients that received percutaneous coronary intervention (PCI); (4) methods for measuring plasma PTX-3 and the cutoff values to define highest and lowest PTX-3 level among the included studies; (5) follow-up durations and related adverse clinical outcomes reported; and (6) potential confounding factors adjusted in the multivariate analyses. The quality of each study was evaluated using the Newcastle–Ottawa Scale (NOS) (32), which ranges from 1 to 9 stars and judges each study with respect to three aspects: selection of the study groups; the comparability of the groups; and the ascertainment of the outcome of interest.

### Statistical Analyses

We used RRs and their corresponding 95% CIs as the general measure for the association between plasma PTX-3 and subsequent incidence of mortality or MACEs in patients with CAD. Data of RRs and their corresponding SEs were calculated from 95% CIs or *p*-values and were logarithmically transformed to stabilize variance and normalized the distribution (31). Cochran's Q test and estimation of *I*^2^ statistic were used to evaluate the heterogeneity among the included cohort studies (33). A significant heterogeneity was considered if *I*^2^ > 50%. We used a random-effects model to synthesize the RR data because this model is considered as a more generalized method, which incorporates the potential heterogeneity among the included studies (31). Sensitivity analyses, by omitting one individual study at a time, were performed to test the robustness of the results (34). Predefined subgroup analyses were performed to evaluate the influences of study characteristics on the outcome including study design characteristics, subtype of CAD, follow-up durations, quality scores, and adjustment of CRP. The medians of continuous variables were used as cutoff values for grouping. The potential publication bias was assessed by visual inspection of the symmetry of the funnel plots as well as Egger's regression asymmetry test (35). If the funnel plots were asymmetrical, a “trim-and-fill” analysis was performed (31). To achieve symmetrical funnel plots, this method assumes the existence of the hypothetically unpublished studies with negative results, estimates their RRs, and recalculates the pooled RR after incorporating this “missing” study (31). A *p* < 0.05 indicates statistical significance. We used the RevMan (version 5.1; Cochrane Collaboration, Oxford, UK) and the STATA software for the meta-analysis and statistics.

## Results

### Literature Search

**Figure 1:**
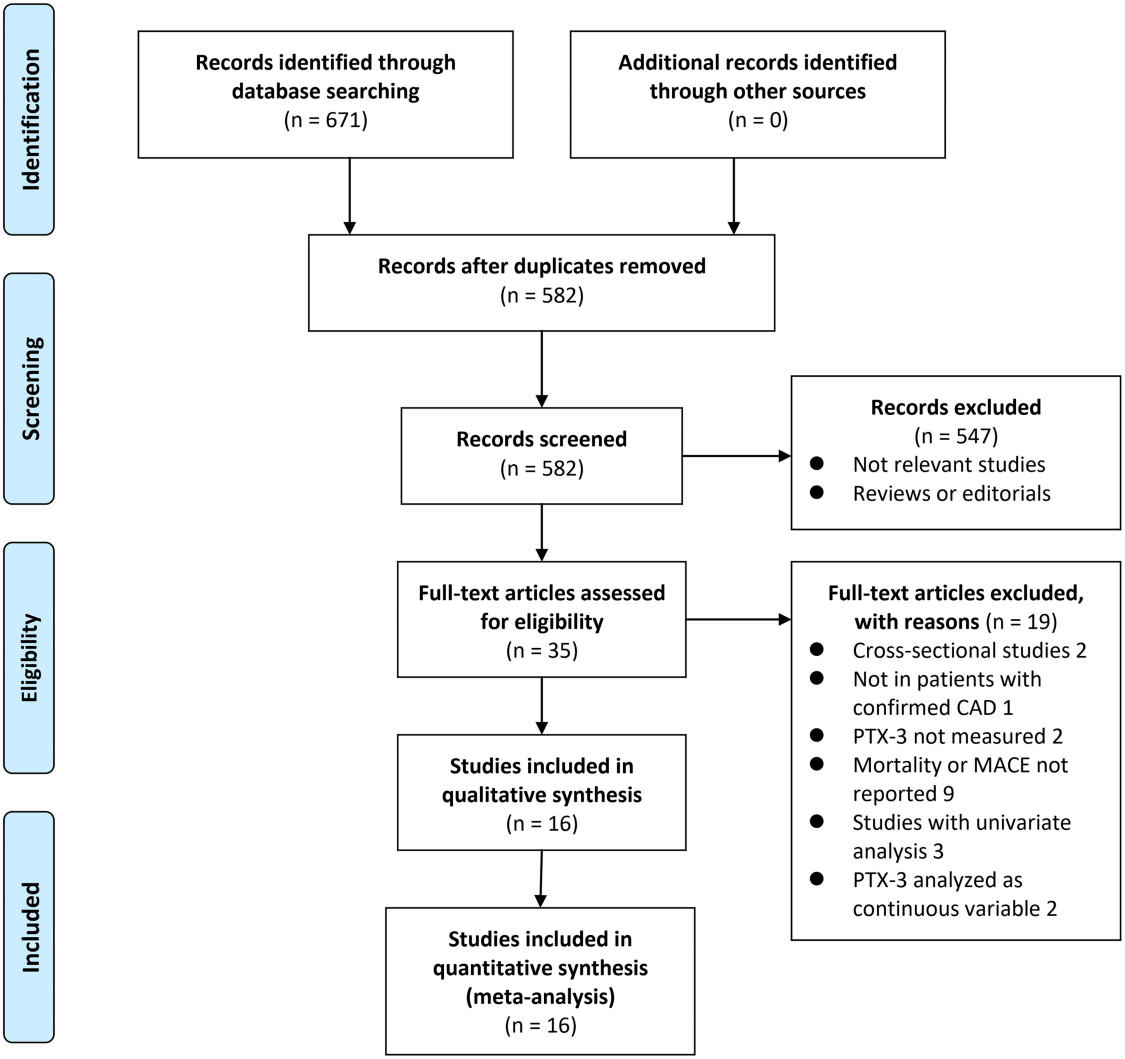
Flowchart of database search and study identification.

The process of database search is given in Figure 1. Briefly, 582 articles were found *via* initial literature search of the PubMed, Embase, and Cochrane Library databases after excluding the duplications. Among them, 547 articles were excluded through screening of the titles and abstracts mainly because they were not relevant to the purpose of the meta-analysis. Subsequently, 35 potential relevant records underwent full-text review. Of these, 19 relevant records were further excluded for the reasons given in Figure 1. Finally, 16 cohort studies were obtained for the meta-analysis (13–28).

### Study Characteristics and Quality Evaluation

The characteristics of the included studies are given in Table 1. Overall, 16 cohort studies including 11,007 adults with CAD were included in the meta-analysis (13–28). These studies were published between 2004 and 2021 and most of the studies were prospective (13–17, 19–28), except one study, which was retrospective (18). For the subtype of CAD, four studies included patients with stable CAD (13, 17, 19, 20), while the other studies included patients with ACS [7 patients with STEMI (14, 21–25, 27), two patients with NSTE-ACS (16, 18), and three patients with both STEMI and NSTE-ACS (15, 26, 28)]. One study reported outcome data in patients with STEMI and NSTE-ACS separately (15) and these datasets were independently included in the meta-analysis. Plasma PTX-3 were measured with ELISA in all of the included studies and various cutoff values were used to compare the incidence of adverse outcomes according to the PTX-3 level such as medians (16, 19, 24, 28), tertiles (13, 14, 17, 21, 23), quartiles (15, 20, 25, 26), and cutoff values derived by measuring of normal controls (18) and from the receiver operating characteristic (ROC) analysis (22, 27). The mean follow-up durations varied between 1 and 60 months. Age, sex, other conventional cardiovascular medications, coronary lesion characteristics, revascularization therapy, and other potential confounding factors were adjusted to a varying degree when the associations between PTX-3 and clinical outcomes were reported. The NOS scores of the included studies ranged from 7 to 9, indicating generally good study quality (Table 1).

**Table 1:** Characteristics of the included cohort studies.

**Study**	**Country**	**Design**	**Patient characteristics**	**Number of patients**	**Mean age**	**Male**	**PCI**	**PTX-3 measuring**	**PTX-3 cutoff**	**Follow-up period**	**Outcomes**	**Variables adjusted**	**NOS**
					**years**	**%**	**%**			**months**			
Latini (14)	Italy	PC	STEMI	724	NR	69.1	4.3	ELISA	T3:T1	3	All-cause mortality and MACEs	Age, sex, smoking, HTN, DM, Killip class, HR, SBP, anterior MI, and CK level	8
Brügger (15)	Norway	PC	STEMI and NSTE-ACS	417	69.5	62.6	NR	ELISA	Q4:Q1	24	All-cause mortality	Age, sex, smoking, HTN, eGFR, DM, HL, HF, history of CAD, peak TnT, BNP and hs-CRP on admission, and CV medications	9
Matsui (16)	Japan	PC	NSTE-ACS	204	69.0	71.0	31	ELISA	Median	6	MACEs	Age, sex, time from symptom onset, AMI, Killip class, emergent PCI, DM, HTN, HL, SBP, HR, hs-CRP, WBC, NT-proBNP, TnI, and eGFR	8
Dubin (17)	USA	PC	Stable CAD	986	66.7	81.5	NR	ELISA	T3:T1	37	All-cause mortality and MACEs	Age, sex, race, DM, HTN, smoking, CRP, and eGFR	9
Guo (18)	China	RC	NSTE-MI	525	57.7	62.5	100	ELISA	Normal control level	1	CV mortality and MACEs	Age, sex, HTN, HL, DM, a history of CAD, smoking, BMI, GFR, SBP, DBP, HR, LVEF, hs-CRP, cTnT, and NT-proBNP	7
Haibo (19)	China	PC	Stable CAD	596	65.9	78.4	100	ELISA	Median	36	MACEs	Age, sex, DM, HTN, HL, current smoking, CCS class, LVEF, hs-CRP, TnI, CV medications, and number and type of stents	9
Miyazaki (20)	USA	PC	Stable CAD	749	60.0	87.0	NR	ELISA	Q4:Q1	60	MACEs	Age, sex, smoking, LVEF, DM, CRP, SBP, DBP, PG, HDL-C, TG, WC, eGFR, and CV medications	9
Akgul (21)	Turkey	PC	STEMI	499	57.1	78.8	100	ELISA	T3:T1-2	24	All-cause mortality	Age, sex, DM, HTN, Killip Class, LVEF, anemia, SCr, and peak TnI	9
Tomandlova (22)	Czech	PC	STEMI	262	63.4	70.6	100	ELISA	ROC analysis derived	12	MACEs	Age, sex, BMI, SBP, HR, SCr, DM, HTN, HL, history of CAD, infarct related artery, and pain onset-balloon time	9
Altay (23)	Turkey	PC	STEMI	140	59.7	72.9	94.6	ELISA	T3:T1	60	CV mortality	Age, sex, hs-CRP, NT-proBNP, GRACE risk score, and LVEF	9
Ljuca (24)	Bosnia and Herzegovina	PC	STEMI	97	61.8	73.2	100	ELISA	Median	24	MACEs	Age, sex, hs-CRP, current smoking, DM, HTN, HL, Killip Class, LVEF, peak TnI, IL-10 and IL-6	9
Dharma (25)	Indonesia	PC	STEMI	335	53.5	85.5	100	ELISA	Q4:Q1	1	All-cause mortality	Age, sex, DM, HTN, anterior MI, SCr, WBC, and PG at admission	8
Zagidullin (27)	Russia	PC	STEMI	147	60.9	80.3	76.2	ELISA	ROC analysis derived	24	CV-mortality	Age, sex, LVEF, peak TnI, ST2 and NT-proBNP	9
Kimura (13)	Japan	PC	Stable CAD	93	69.0	69.9	100	ELISA	T3:T1-2	9	MACEs	Age, sex, BMI, current smoking, DM, HTN, HL, previous MI, LVEF, CV medications, and coronary lesion features	8
Kontny (26)	Sweden	PC	ACS	5154	62.0	70.1	61.0	ELISA	Q4:Q1	12	CV-mortality and MACEs	Age, sex, DM, CHF, type of ACS, in-hospital treatment approach, previous PCI/CABG/MI/ PAD/non-hemorrhagic-stroke, CKD, HTN, smoking status, HL, and BMI	8
Jiang (28)	China	PC	ACS	79	63.7	51.9	NR	ELISA	Median	3	MACEs	Age, sex, hs-CRP, DM, HTN, HL, CTRP9 and peak TnI	8

*PTX-3, pentraxin-3; PCI, percutaneous coronary intervention; NOS, Newcastle–Ottawa Scale; PC, prospective cohort; RC, retrospective cohort; CAD, coronary artery disease; ACS, acute coronary syndrome; STEMI, ST-segment elevation myocardial infarction; NSTE-ACS, non-ST-segment elevation ACS; NSTE-MI, non-ST-segment elevation myocardial infarction; NR, not reported; T, tertile; Q, quartile; ROC, receiver operating characteristic; MACEs, major adverse cardiovascular events; CV, cardiovascular; HTN, hypertension; DM, diabetes mellitus; BMI, body mass index; SBP, systolic blood pressure; DBP, diastolic blood pressure; HR, heart rate; HF, heart failure; HL, hyperlipidemia; WBC, while blood cell count; hs-CRP, high-sensitivity C-reactive protein; BNP, B-type natriuretic peptide; NT-proBNP, N-terminal pro-BNP; eGFR, estimated glomerular filtration rate; LVEF, left ventricular ejection fraction; TnT, troponin T; TnI, troponin I; CCS, Canadian Cardiovascular Society; PG, plasma glucose; WC, waist circumference; HDL-C, high-density lipoprotein cholesterol; SCr, serum creatinine; GRACE, Global Registry of Acute Coronary Events; IL-10, interleukin-10; IL-6, interleukin-6; CABG, coronary artery bypass grafting; PAD, peripheral artery disease; CTRP9, C1q/tumor necrosis factor (TNF)-related protein 9*.

### Association Between PTX-3 and Mortality Outcomes in Patients With CAD

**Figure 2:**
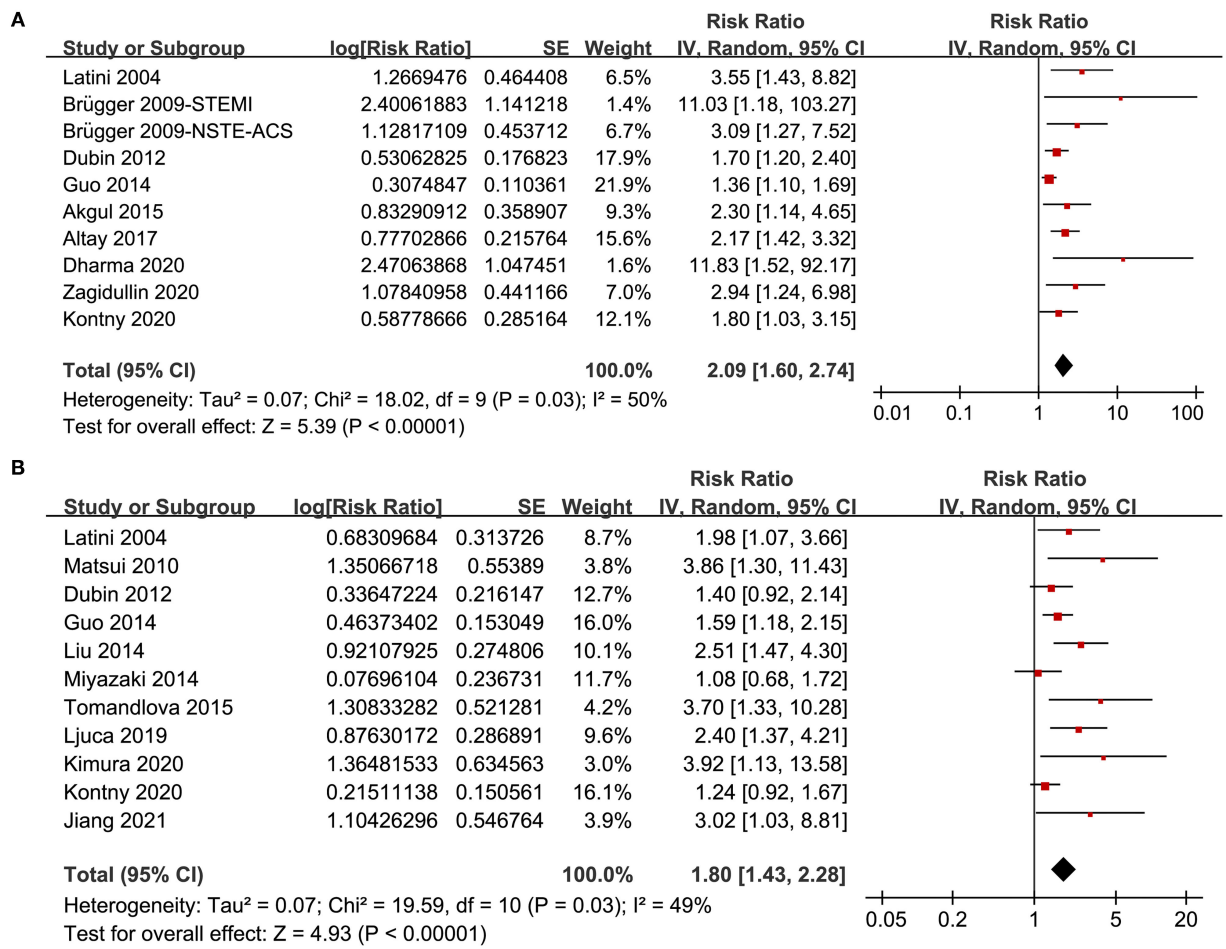
Forest plots for the meta-analysis of the association between pentraxin-3 (PTX-3) and clinical outcomes in patients with coronary artery disease (CAD); **(A)** forest plots for the association between PTX-3 and mortality in patients with CAD and **(B)** forest plots for the association between PTX-3 and major adverse cardiovascular events (MACEs) in patients with CAD.

A total of 10 datasets from 9 studies (14, 15, 17, 18, 21, 23, 25–27) reported the association between PTX-3 level and mortality risk in patients with CAD. Pooled results showed that compared to patients with the lowest PTX-3 level, those with the highest PTX-3 level had significantly increased risk of mortality (adjusted RR: 2.09, 95% CI: 1.60 to 2.74, *p* < 0.001; Figure 2A) with moderate heterogeneity (*I*^2^ = 50%, *p* for Cochran's Q test = 0.03). Sensitivity analysis by omitting one study at a time showed consistent results (RR: 1.99–2.29, *p* < 0.01). Besides, predefined subgroup analysis showed consistent results in studies of different designs (prospective or retrospective), subtypes of CAD (STEMI, NSTE-ACS, or stable CAD), follow-up duration (within 1 year or over 1 year), definitions of mortality outcome (all-cause mortality or cardiovascular mortality), different quality scores, and with or without adjustment of CRP (*p*-values for subgroup effects < 0.05; Table 2).

**Table 2:** Subgroup analyses for the association between PTX-3 and mortality in patients with CAD.

	**Association between PTX-3 and mortality in CAD patients**				
**Study characteristics**	**Datasets number**	**RR (95% CI)**	***I* ^2^**	***P* for subgroup effect**	***P* for subgroup difference**
**Study design**					
PC	9	2.21 [1.75, 2.81]	13%	<0.001	
RC	1	1.36 [1.10, 1.69]	—	0.005	0.003
**Diagnosis**					
STEMI	6	2.59 [1.90, 3.52]	0%	<0.001	
NSTE-ACS	2	1.42 [1.15, 1.76]	68%	0.001	
Stable CAD	1	1.70 [1.20, 2.40]	—	0.003	0.007
**Follow-up durations**					
Within 1 year	3	1.46 [1.19, 1.80]	75%	<0.001	
More than 1 year	7	2.04 [1.65, 2.53]	0%	<0.001	0.03
**Outcomes reported**					
All-cause mortality	6	2.65 [1.68, 4.17]	40%	<0.001	
CV mortality	4	1.78 [1.29, 2.46]	52%	<0.001	0.16
**Study quality**					
NOS 7~8	4	2.07 [1.19, 3.62]	65%	0.01	
NOS = 9	6	2.09 [1.66, 2.63]	0%	<0.001	0.98
**Adjustment of CRP**					
Yes	5	1.84 [1.33, 2.53]	57%	<0.001	
No	5	2.47 [1.70, 3.57]	6%	<0.001	0.24

*RR, risk ratio; PC, prospective cohort; RC, retrospective cohort; NOS, Newcastle–Ottawa Scale; STEMI, ST-segment-elevation myocardial infarction; NSTE-ACS, non-ST-segment-elevation acute coronary syndrome; CAD, coronary artery disease; PTX-3, pentraxin-3; CV, cardiovascular; CRP, C-reactive protein*.

### Association Between PTX-3 and MACEs in Patients With CAD

A total of 11 studies (13, 14, 16–20, 22, 24, 26, 28) reported the association between PTX-3 incidence of MACEs in patients with CAD. Pooled results showed that higher PTX-3 was independently associated with a higher risk of MACEs in patients with CAD during follow-up duration (RR: 1.80, 95% CI: 1.43 to 2.28, *p* < 0.001; Figure 2B) with moderate heterogeneity (*I*^2^ = 49%, *p* for Cochran's Q test = 0.03). Sensitivity analysis by omitting one study at a time retrieved similar results (RR: 1.73–1.93, *p* < 0.01). In addition, results of predefined subgroup analysis showed consistent results in studies of different study designs, subtype of CAD, follow-up duration, quality scores, and with or without adjustment of CRP (*p*-values for subgroup effects < 0.05; Table 3).

**Table 3:** Subgroup analyses for the association between PTX-3 and MACEs in patients with CAD.

	**Association between PTX-3 and MACEs in CAD patients**				
**Study characteristics**	**Datasets number**	**RR (95% CI)**	***I* ^2^**	***P* for subgroup effect**	***P* for subgroup difference**
**Study design**					
PC	10	1.90 [1.43, 2.52]	54%	<0.001	
RC	1	1.59 [1.18, 2.15]	—	0.002	0.40
**Diagnosis**					
STEMI	3	2.37 [1.61, 3.48]	0%	<0.001	
NSTE-ACS	2	1.69 [1.27, 2.26]	58%	<0.001	
Stable CAD	4	1.55 [1.19, 2.02]	61%	0.001	0.20
**Follow-up durations**					
Within 1 year	7	1.94 [1.40, 2.69]	49%	<0.001	
More than 1 year	4	1.69 [1.14, 2.52]	62%	0.01	0.61
**Study quality**					
NOS 7~8	6	1.79 [1.30, 2.46]	45%	<0.001	
NOS = 9	5	1.84 [1.24, 2.74]	61%	0.003	0.91
**Adjustment of CRP**					
Yes	6	1.71 [1.26, 2.32]	48%	<0.001	
No	5	2.06 [1.32, 3.23]	60%	0.002	0.50

*RR, risk ratio; PC, prospective cohort; RC, retrospective cohort; NOS, Newcastle–Ottawa Scale; STEMI, ST-segment-elevation myocardial infarction; NSTE-ACS, non-ST-segment-elevation acute coronary syndrome; CAD, coronary artery disease; PTX-3, pentraxin-3; MACEs, major adverse cardiovascular events; CRP, C-reactive protein*.

### Publication Bias

**Figure 3:**
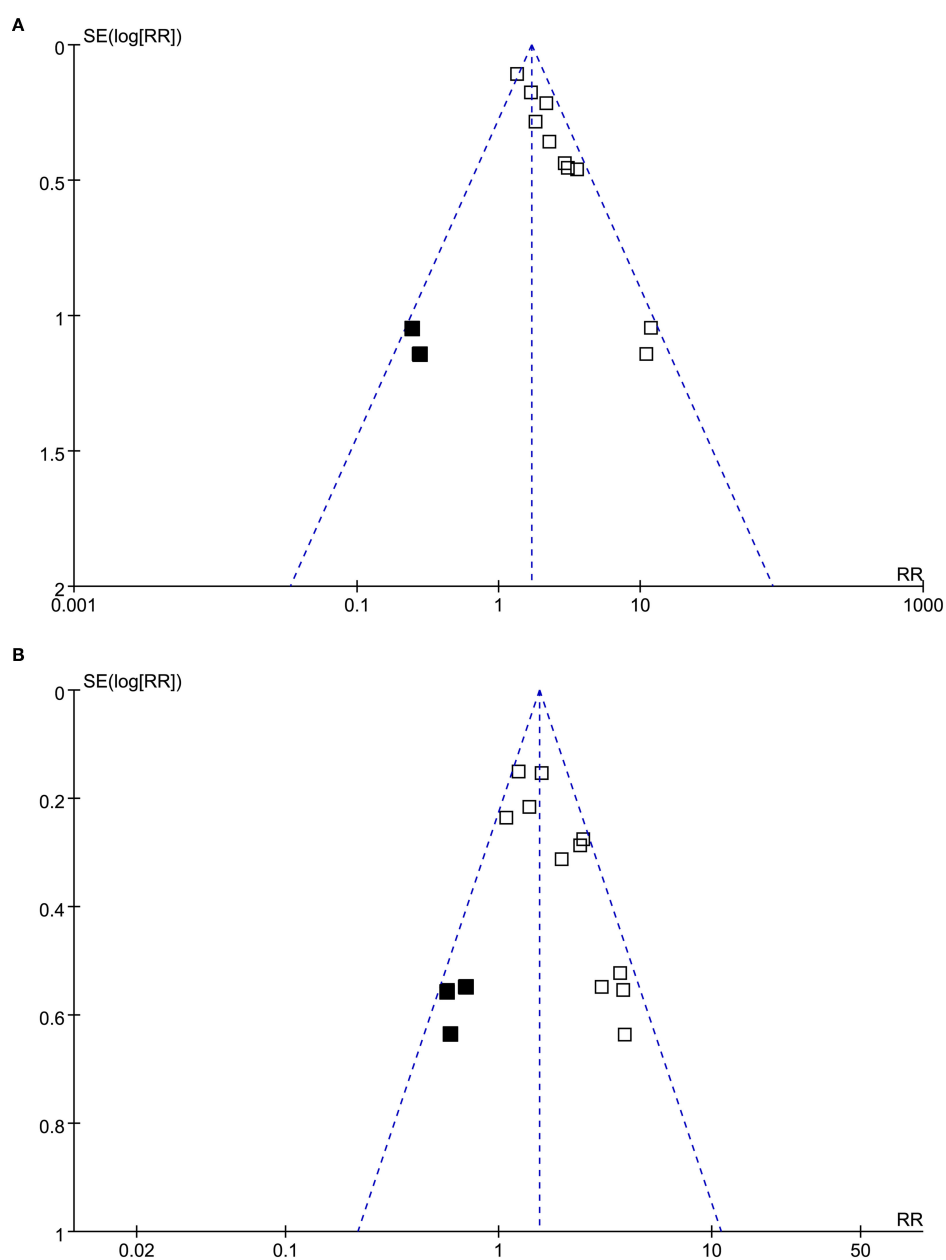
Funnel plots for the publication bias underlying the meta-analyses; **(A)** funnel plots with trim-and-fill analysis for the meta-analysis of the association between PTX-3 and mortality in patients with CAD and **(B)** funnel plots with trim-and-fill analysis for the meta-analysis of the association between PTX-3 and MACEs in patients with CAD. The black square indicates hypothesized studies to achieve the symmetry of the funnel plots.

The funnel plots with respect to the associations between PTX-3 and mortality and MACEs are shown in Figures 3A,B separately, which were asymmetry on visual inspection. Results of Egger's regression test also suggested the potential risks of publication bias (*p*-values = 0.082 and 0.045, respectively). We, therefore, performed a trim-and-fill analysis. As shown in Figures 3A,B, incorporating the hypothesized studies (indicated by black squares, two studies for the outcome of mortality, and three studies for the outcome of MACEs) achieved the symmetry of funnel plots and the results of meta-analysis remained significant after including these hypothesized studies (mortality outcome: RR: 2.00, 95% CI: 1.50 to 2.68, *p* < 0.001; MACEs outcome: RR: 1.64, 95% CI: 1.28 to 2.09, *p* < 0.001).

## Discussion

In this meta-analysis, by pooling the results of 16 cohort studies, we found that compared to patients with CAD with the lowest level of circulating PTX-3, those with the highest level of PTX-3 had significantly increased risk of mortality and MACEs during follow-up. Further sensitivity analysis by excluding one study at a time did not significantly change the results, suggesting the robustness of the findings. In addition, multiple predefined subgroup analyses also showed that the association between higher PTX-3 and poor prognosis was consistent in prospective and retrospective studies, in patients with stable CAD and ACS, in studies with short- and long-term follow-up durations, in studies with different quality scores, and in those with and without adjustment of CRP. Although potential publication biases were suggested, further trim-and-fill analysis by incorporating the hypothesized studies to achieve symmetrical funnel plots showed similar results. Taken together, results of the meta-analysis indicated that higher plasma PTX-3 is associated with poor prognosis in patients with CAD, which may be independent of the CAD subtype, follow-up durations, and adjustment of CRP.

A total of one previous meta-analysis has been published to summarize the association between PTX-3 and clinical outcomes in patients with CAD (29). However, due to the limited studies included (two to five studies for each outcome), the authors failed to show that higher PTX-3 is associated with increased risk of cardiac events in patients with stable CAD (29). Moreover, they were also unable to explore the potential influences of the patient or study characteristics on the results of the meta-analyses (29). Compared to the previous one, this study has a few strengths. Firstly, only cohort studies with multivariate analysis were included, which, therefore, could provide an independent association between higher PTX-3 and poor prognosis in patients with CAD. In addition, an extensive and updated literature search was performed. We identified a total of 16 cohort studies and the relative large number of datasets enabled us to perform comprehensive sensitivity and subgroup analyses. The overall results of the meta-analysis showed that higher PTX-3 is associated with increased risk of mortality and MACEs in patients with CAD. Different from the results of the previous meta-analysis, we found that the association between higher PTX-3 and poor prognosis was not only limited in patients with stable CAD, but also in patients with ACS. Moreover, consistent results were obtained in patients with STEMI and in those with NSTE-ACS. Results of additional subgroup analyses also showed consistent results in studies with different designs, follow-up durations, quality scores, and in studies with and without adjustment of CRP. These results further confirmed a stable role of higher PTX-3 as a predictor of poor prognosis in patients with different spectrum of CAD.

The potential mechanisms underlying the association between higher PTX-3 and increased risks of morality and adverse events in patients with CAD remain not fully understood. It has been shown that higher PTX-3 could predict the risk of heart failure (HF) in patients with ACS (36). Besides, higher PTX-3 has been correlated with the severity of coronary lesions and vulnerability of coronary plaque components in patients with stable CAD (13, 37–40) and ACS (41). In addition, compared to CRP, PTX-3 may be a more sensitive marker of systematic inflammation (42) and a more potent predictor of endothelial dysfunction (43), both of which have been involved in the deterioration of coronary lesions. Besides, an early study showed that although PTX-3 and CRP were more enhanced in unstable than in stable coronary plaques, their distribution distinctly differed, suggesting that they play distinct biological roles in unstable plaques (44). More experimental studies are needed to determine whether increased circulating PTX-3 plays an important role in the progression of CAD or it is simply a marker of increased atherosclerotic burden.

This study also has some limitations. First, the meta-analysis was based on data from the study level rather than individual patients, which prevented further analyses on the influences of the characteristics of the patient on the outcome such as age, sex, comorbidities, and concurrent medications. Particularly, statins have been proposed to reduce the level of circulating PTX-3 in previous studies (45, 46), which may affect the association between PTX-3 and poor prognosis in patients with CAD. In addition, the optimized cutoff values of circulating PTX-3 for predicting poor prognosis in patients with different subtypes of CAD remain to be determined. Moreover, it remains unknown whether incorporation of PTX-3 into the current risk stratification tool for patients with CAD is associated with improved predictive efficacy. For example, it is currently unknown that for patients with ACS, whether measuring of PTX-3 still has prognostic significance on the basis of currently established prognostic models such as the Global Registry of Acute Coronary Events (GRACE) scoring system. Studies are needed to address this issue. Finally, publication bias was suggested by the results of Egger's regression test for the meta-analysis of the association between higher PTX-3 and poor prognosis in CAD. However, further trim-and-fill analysis suggested that the potential publication bias was not likely to affect the overall findings of the meta-analysis.

In conclusion, results of the meta-analysis suggest that higher plasma PTX-3 is associated with poor prognosis in patients with CAD, which may be independent of the CAD subtype, follow-up durations, and adjustment of CRP. Future studies are needed to elucidate the potential pathological role of PTX-3 in the pathogenesis and progression of CAD. Besides, whether measuring PTX-3 could improve risk stratification in patients with CAD, particularly in those with ACS, should also be evaluated in the future.

## Data Availability Statement

The original contributions presented in the study are included in the article/supplementary material, further inquiries can be directed to the corresponding author.

## Author Contributions

KD and XY designed the study. KD and ZS performed database search, study identification, data extraction, and study quality evaluation. KD, ZS, and CQ performed statistical analysis and interpreted the results. KD and XY drafted the manuscript. All the authors revised the manuscript and approved the submission of the manuscript.

## Funding

This study was supported by the Zhejiang Provincial Medical and Health Science and Technology Project (Nos. 2019329308 and 2020371419), the Shaoxing Science and Technology Plan Project (No. 2020A13084), and the Zhuji Medical and Health Science and Technology Project (No. 2019YW051).

## Conflict of Interest

The authors declare that the research was conducted in the absence of any commercial or financial relationships that could be construed as a potential conflict of interest.

## Publisher's Note

All claims expressed in this article are solely those of the authors and do not necessarily represent those of their affiliated organizations, or those of the publisher, the editors and the reviewers. Any product that may be evaluated in this article, or claim that may be made by its manufacturer, is not guaranteed or endorsed by the publisher.
